# Optimization of an *in vitro* assay methodology for competitive binding of thyroidogenic xenobiotics with thyroxine on human transthyretin and albumin

**DOI:** 10.1016/j.mex.2017.10.004

**Published:** 2017-10-20

**Authors:** Katie L. Hill, Timo Hamers, Jorke H. Kamstra, William G. Willmore, Robert J. Letcher

**Affiliations:** a Ecotoxicology and Wildlife Health Division, Environment and Climate Change Canada, National Wildlife Research Centre, Carleton University, Ottawa, Canada; b Department of Biology, Carleton University, Ottawa, Canada; c Intrinsik Corp., Ottawa, Canada; d Department of Environment and Health, Vrije Universiteit Amsterdam, The Netherlands; e Faculty of Veterinary Medicine and Biosciences, Department of Basic Science and Aquatic Medicine, CoE CERAD, Norwegian University of Life Sciences, 0033 Oslo, Norway

**Keywords:** Thyroid hormones, Transport proteins, Transthyretin, Albumin, Human, Competitive protein binding assay, Exogenous ligands

## Abstract

Thyroid hormones (THs) are involved in the regulation of many physiological processes in vertebrates. Competition for TH binding sites on serum transport proteins can interfere with delivery of THs to target tissues, and this is a potential mechanism of action of exogenous thyroidogenic substances. To date, detailed accounts of *in vitro* methods for competitive binding with THs on TH transport proteins (human or wildlife) are sparse. In the limited number of published studies on *in vitro* radio-labelled TH-TH transport protein interactions, method descriptions were brief and with insufficient details for successful replication. Furthermore, upon review of these methodologies, we identified several opportunities for optimization. The present study addresses the methodological deficiencies and describes, in detail, a fully optimized and validated competitive T4 radio-ligand binding assay with human transthyretin (TTR) and albumin (ALB).

• Significant improvements were made over previous methods, including better maintenance of protein stability and enhanced measurement of competition between different ligands.
• Sample size was reduced to allow use of small pre-packed size exclusion chromatography columns, which eliminates the rinsing step during the separation procedure.
• The assay was parameterized for use with T4 and human TTR and ALB.

## Graphical abstract

**Figure:**
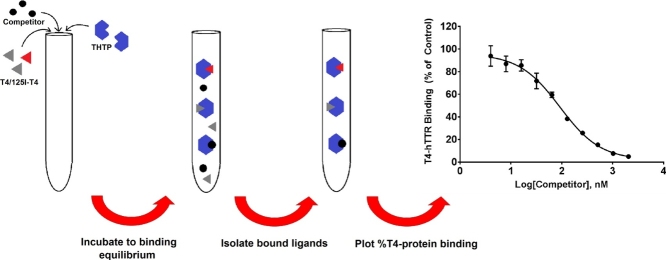

## Method details

### Background

The structural resemblance of many anthropogenic chemicals to thyroid hormones (THs) warrants concern for the perturbation of TH-dependent processes in humans and other vertebrates [1]. Thyroxine (T4) and 3,5,3-triiodothyronine (T3) are THs involved in the regulation of many important physiological processes in the body including neurological and behavioural development, growth, metabolism, and respiration [2]. In mammals (including humans), circulating T4 concentrations are greater in plasma than for T3. T4 is the prohormone TH, and it is deiodinated via enzyme-controlled mechanisms to T3, which is the main ligand for the TH receptor leading to gene regulation [3]. The vast majority (>99%) of THs in plasma are delivered to target tissues by binding to TH transport proteins, which include albumin (ALB), transthyretin (TTR), and thyroxine-binding globulin (TBG). The TH binding affinities and dissociation constants of these three proteins vary, with ALB having the lowest relative affinity for T4 and the greatest concentration in plasma; and TBG having the highest affinity for T4, and the lowest concentration in plasma [4]. TTR is the only TH transport protein synthesized in the choroid plexus, and it thus plays a role in the movement of THs from the blood into the cerebrospinal fluid (CSF) which is particularly important in early development [3].

One potential mechanism of action of xenobiotic thyroidogenic substances is competition for TH binding sites on serum transport proteins, as this can interfere with delivery of THs to target tissues. A limited number of previously published studies report variations of the *in vitro* radiolabelled T4-TTR binding assay technique, with target chemicals including polychlorinated biphenyls, perfluorinated compounds, polybrominated diphenyl ethers, and some degradation products [1], [5], [6], [7], [8], [9], [10]. However, the methods reported in these studies do not provide sufficient details to successfully replicate the procedure and we faced several challenges in attempting to do so. Therefore, the objectives of the present study were to refine, optimize, validate, and fully describe a competitive *in vitro* radio-ligand-protein binding assay that is used to investigate thyroidogenicity of chemicals via interaction between T4 and human TTR. Furthermore, we expanded this method to include interactions between T4 and human ALB which, to our knowledge, has not been adequately described elsewhere. The model xenobiotic chemical that we used as a competitor ligand is 4-hydroxy-2,2′,4,5′-tetrabromodiphenyl ether (4-OH-BDE-49), which is often detected in human serum [11] and has previously been found to bind to human TTR isolated from human plasma with higher affinity than T4 *in vitro* [9].

A number of other methods have been developed for the purpose of investigating potential competition of xenobiotics with T4 for TTR, including the ANSA (8-anilino-1-naphthalenesulfonic acid ammonium)-TTR competitive fluorescence displacement assay [12], the TR-CALUX (thyroid hormone responsive chemically activated luciferase gene expression) assay [13], surface plasmon resonance-based biosensor assays [14], FITC-T4 (fluorescence probe fluorescein isothiocyanate associated to T4) assays [15], [16], and HPLC (high performance liquid chromatography) assays [17]. Notably, Ouyang et al. [16] presents an enhanced FITC-T4 assay that has been miniaturized into a 96 well microplate to generate a high throughput method. While the miniaturized FITC-T4 method offers increased efficiency, the radiolabelled T4-protein binding assay described herein is more sensitive than the assay presented in Ouyang et al. [16], and by an order of magnitude for potent xenobiotic competitors (those with IC50s < 100 nM). This is particularly important for the detection of receptor binding events at low concentrations (e.g., those found in blood or other biological samples) and for reducing the likelihood of a type II error. Additionally, our method extends to include interactions with ALB, which increases the relevance of this assay as ALB is the most abundant protein in plasma and serum.

### Sample preparation (Day 1)

**Fig. 1:**
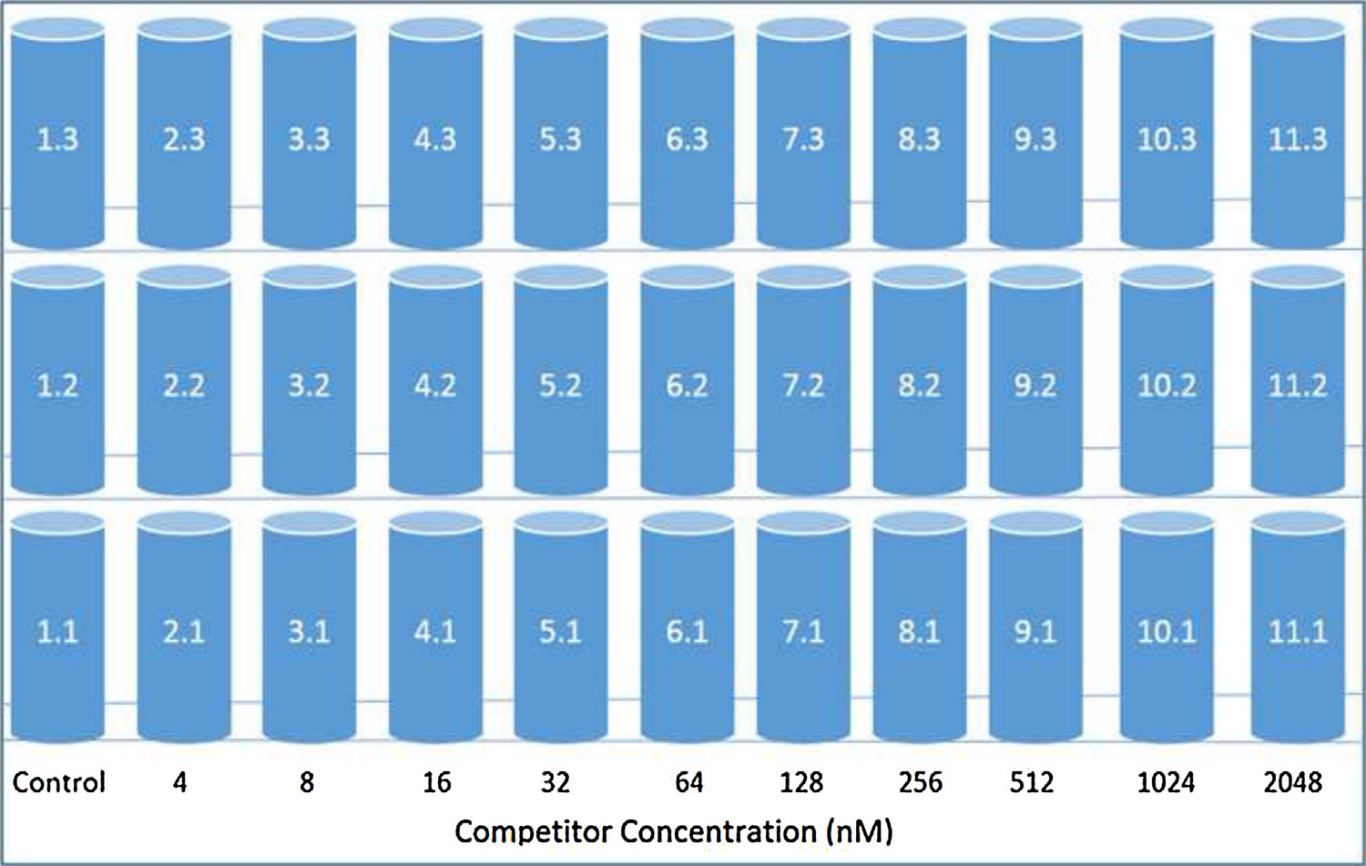
Example of the sample set up procedure, including vial labels and concentrations of thyroxine (T4) as a competitor ligand for the calibration exercise.

The entire assay takes two days to complete for each batch of samples to be processed. The Day 1 procedure is described as follows. Prepare the solutions of the proteins, THs and competitive ligands to be used in the *in vitro* assay according to Table 1 and using materials specified in Table 2. First, thaw the working solutions of these components to room temperature if they have been placed in frozen storage. For the protein working solutions, prepare the day they are to be used based on the number of assay aliquots required (one vial is sufficient for approximately 45 assay samples). Take note of the protein handling recommendations in the Precautions and Tips section. Label a series of polypropylene test tubes corresponding to the samples being prepared. This should include triplicate samples for each ligand competitor concentration. See Fig. 1 for an example of the sample set up for a batch of assays including vial labels and contents.

**Table 1:** Preparation and storage details for the stock and final concentrations of the ligands and transport proteins for the thyroid hormone competitive binding assay.

Component	Stock Solution			Working Solution			Final Concentration in Sample
	Concentration	Preparation	Storage	Concentration	Preparation	Volume Added to Sample	
Protein (Select One)							
TTR	3.64 μM	Dissolve 0.5 mg into 2500 μL Tris-EDTA by inversion and portion into 40 μL aliquots	1500 μL polypropylene microcentrifuge tubes at −20 °C for up to one year	120 nM	Add 1160 μL Tris-EDTA to aliquot on day of use	25 μL	30 nM
ALB	72.8 μM	Dissolve 12.1 mg into 2500 μL Tris-EDTA by inversion and portion into 40 μL aliquots	1500 μL pp tubes at −20° C for up to one year	2400 nM	Add 1160 μL Tris-EDTA to aliquot on day of use	25 μL	600 nM

Natural Ligand							
T4	1000 μM	Dissolve 5 mg into 6435 μL DMSO in a test tube by gentle vortex and transfer to storage vessels	1500 μL pp tubes at −20 °C for up to three years	11,000 nM	Add 11 μL stock solution to 989 μL DMSO	70 μL (0.5 μL T4, 5 μL ^125^I-T4, and 64.5 μL Tris-EDTA per sample)^a^	55 nM + ∼5%^b^
^125^I-T4	0.0536 μM (1000 μCi/g)	Transfer contents of ampoule to storage vessel	1500 μL pp tube at 4 °C for up to two months	(use stock)			

Competitor Ligand (Select One)							
T4	(see above)			20 to 40,960 nM	Perform two-fold dilution series from 40,960 to 20 nM using T4 stock to prepare standards for calibration curve	5 μL	1 to 2048 nM^c^
4-OH-BDE-49	19.9 μM	Conduct a solvent exchange for DMSO using nitrogen evaporator	2000 μL amber glass vials at 4° C for several years	5 to 5120 nM	Perform two-fold dilution series from 5 to 5120 nM	5 μL	0.125 to 256 nM^d^
						Total Volume Per Sample = 100 μL	

^a^See Sample Preparation for details.

^b^Concentration of ^125^I-T4 varies depending on decay schedule (see Precautions and tips).

^c^Concentration range of 1 to 2048 nM for TTR; 1 to 1024 nM for ALB.

^d^Concentration range of 0.25 to 256 nM for TTR; 0.125 to 128 nM for ALB.

**Table 2:** Description of the reagents, materials and equipment required to conduct the thyroid hormone competitive binding assay.

Component	Details	Source
Reagents		
Transthyretin (TTR)	Lyophilized from human plasma (≥95%)	Sigma-Aldrich P1742
Albumin (ALB)	Lyophilized from human plasma (≥99%)	Sigma-Aldrich A8763
L-thyroxine (T_4_)	(≥98%)	Sigma-Aldrich T2376
Radio-labeled L-thyroxine (^125^I-T_4_)	50 μCi (800–1000 μCi/g)	MP Biomedicals 07190128
4′-hydroxy-2,2′,4,5′-tetrabromodiphenyl ether (4-OH-BDE-49)	10 μg/mL in acetonitrile (97.8%)	Chromatographic Specialties Inc. AHBDE4002SCN02X
Tris-EDTA buffer solution	10 mM Tris–HCL, 1 mM EDTA, pH 8.0	Sigma-Aldrich 93283

Materials		
Amber glass vials	2 mL with lids	Chromspec C779100A
Eppendorf^®^ LoBind microcentrifuge tubes	1.5 mL with attached caps	Sigma-Aldrich Z666505
Polypropylene test tubes	12 × 75 mm with lids	Sigma-Aldrich T1911
Bio-Spin^®^ P-6 Gel Columns	Tris buffer, sample volume 50–100 μL	Bio-Rad 732-6228

Equipment		
Eppendorf^®^ pipettes various sizes with corresponding tips		
Nitrogen evaporator		
Fridge (4 °C) and freezer (−20 °C)		
Temperature controlled centrifuge (4 °C)		
Gamma counter in radioactivity-licensed laboratory		
Analytical balance		

Prepare the T4/^125^I-T4/Tris-EDTA buffer solution using one polypropylene test tube per triplicate set of samples. For each, pipette 20 μL of T4 working solution, 200 μL of ^125^I-T4 stock solution (see Precautions and tips for details on signal strength required), and 2580 μL of Tris-EDTA into the test tube(s) and invert to mix (resulting in the addition of 0.5 μL T4, 5 μL ^125^I-T4, and 64.5 μL Tris-EDTA to each sample).

The following steps describe the order of pipetting components for assay incubation mixtures.

1. Starting with the calibration curve samples (T4 as a competitor), pipette 5 μL of the lowest competitor concentration into all replicate assays. Continue for each increasing concentration of the ligand. Control samples receive 5 μL of DMSO in place of a competitor ligand.
2. For the first “replicate set” (e.g., 1.1–11.1 in Fig. 1), pipette 70 μL of the T4/^125^I-T4 solution into each test tube, followed by 25 μL of the selected protein (e.g., TTR). This results in a total sample volume of 100 μL.
3. Centrifuge the samples at 300 × *g* for 30 s to force the solution to the bottom of the tube.
4. Measure the initial radioactivity on the gamma counter. Cap the samples immediately, being careful not to disturb solutions, and place in the fridge at 4 °C overnight to reach protein-ligand binding equilibrium.

While the first replicate set is on the gamma counter, repeat Steps 2 to 4 for the subsequent replicate set(s) (e.g., 1.2 to 11.3). Follow the same preparation sequence for the xenobiotic competitor(s) being analyzed (e.g., 4-OH-BDE-49).

### Separation procedure (Day 2)

Label a series of clean test tubes corresponding to the samples prepared on Day 1 and repeat these labels on Bio-Spin^®^ columns. Work on one replicate set at a time, leaving the remaining columns and incubation mixtures in the fridge until ready for processing. Prepare spin columns for use according to Bio-Spin^®^ column instructions (invert column to re-suspend gel, remove cap and tip, centrifuge in test tube at 1000 × *g* for 2 min, discard drained packing buffer). Process the incubation mixtures as follows:

1. Extract the first sample of the replicate set from the incubation tube using a 100 μL pipette and load the sample to the head of the corresponding column, being careful not to disturb the gel bed. Release the emptied pipette tip into the incubation tube.
2. Repeat for each sample in the replicate set, and centrifuge samples for 4 min at 1000 × *g*.
3. Measure the radioactivity of the eluate fraction, as well as the residual radioactivity in the incubation tubes containing the discarded pipette tips.

Repeat Steps 1 to 3 for each of the remaining replicate sets. The separation procedure involves size exclusion chromatography, whereby the protein-bound ligand is eluted from the Bio-Spin^®^ columns and unbound ligand remains in the columns. The size exclusion limit of the chromatography columns is 6000 g/mol, and thus any compound smaller than this size will travel through the polyacrylamide beads (a longer route through the column) while larger molecules will move around the beads (a shorter route through the column). Measuring the radioactivity of the emptied incubation tubes and pipette tips accounts for nonspecific binding and any potential transfer loss.

*Note*: Previous versions of this method [1], [8], [9], [10] suggest an additional step involving rinsing of the columns with Tris buffer, followed by a second round of centrifugation to elute all protein-bound ligand. This step was found to be unnecessary as the present method is optimized to the sample volumes and Bio-Spin^®^ columns used.

### Precautions and tips

TTR is a labile substance and is susceptible to denaturation. It is thus important to handle proteins with care when preparing solutions and processing samples to ensure they remain intact. Do not vortex solutions containing TTR. Prepare working solutions of TTR or ALB only on the day of use to ensure that the protein structural integrity is maintained. Additionally, keep solutions containing proteins on ice during sample preparation and processing also to prevent denaturing. Incubation mixtures should be left in the fridge until ready for processing, and analyzed quickly so as to reduce time on the column. Use a temperature controlled centrifuge set to 4° C to maximize protein integrity. Furthermore, it is recommended that Eppendorf LoBind^®^ Polypropylene test tubes be used to prepare incubation samples, in order to minimize potential loss of proteins and ligands from adsorption to the vessel.

Care must also be taken when handling radiolabelled T4. All components of the assay involving ^125^I-T4 must be completed in a radioactivity-licensed laboratory by trained personnel. Wear the necessary personal protective equipment, and work under a fume hood for all steps involving pipette work. The proportion of ^125^I-T4 added to samples can be varied depending on signal strength, aiming for >10,000 cpm per sample for initial readings. Adjust the amount of Tris-EDTA added to each sample accordingly.

### Data analysis

**Fig. 2:**
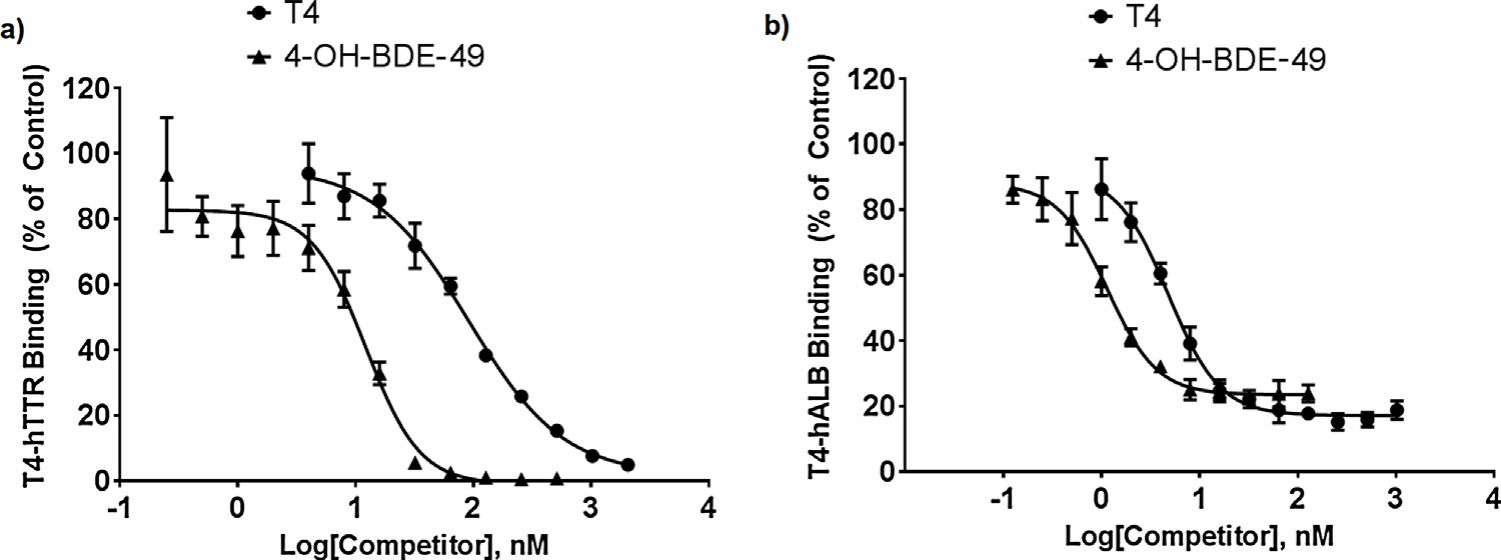
Competitive ligand binding curves for (A) thyroxine (T4)-transthyretin (TTR) interactions with T4 or with the competitor ligand 4-OH-BDE-49, and (B) T4-albumin (ALB) interactions with T4 or with competitor ligand 4-OH-BDE-49. Results are presented as relative percent of T4 bound to protein compared to controls (means ± standard deviations; 6 replicates for each concentration tested).

Calculate the percent of protein binding for each sample by dividing the radioactivity of eluate on Day 2 by the initial radioactivity measured on Day 1, minus any transfer loss (i.e., % binding = eluate cpm/[Day 1 cpm − cpm of emptied incubation tube and pipette tip]). Express results with the logarithmic competitor concentration on the X-axis, and mean ± standard deviation of percent binding compared to controls on the Y-axis (Fig. 2). Generate IC50 estimates in GraphPad Prism 7.02 (GraphPad Software, Inc., La Jolla, CA), using the [inhibitor] vs. response − variable slope equation (Table 3), defined as:

Y = Bottom + (Top − Bottom)/(1 + 10^(LogEC50-X)*Hillslope^)

The inhibition constant (K_i_) for each competitor can be determined based on Cheng and Prusoff (1973) and following GraphPad guidance [18], [19]. In a homologous assay (i.e., with T4 as the competitor) the cold ligand and radioligand can be assumed to have the same binding affinities. As such, the K_i_ and the equilibrium dissociation constant (K_d_) for T4 are determined using the IC50 of T4 and the concentration of radioligand in samples:

(K_i_)_T4,125I-T4_ = (K_d_)_T4,125I-T4_ = (IC50)_T4_ − [^125^I-T4]

With these parameters defined, the K_i_ of other competitors can be determined using the following equation:

(K_i_)_competitor_ = (IC50)_competitor_/(1 + [^125^I-T4]/(K_d_)_125I-T4_)

**Table 3:** IC50 and K_i_ values for thyroxine (T4) and 4-OH-BDE-49 competitive binding to the human thyroid hormone transport proteins transthyretin (TTR) or albumin (ALB).

Competitor	T4-TTR		T4-ALB	
	IC50 (nM)	K_i_	IC50 (nM)	K_i_
T4	91.5	91.2	4.80	4.52
4-OH-BDE-49	12.2	12.2	1.15	1.08

A simplified method for calculating K_i_ is as follows:

(IC50)_T4_/(IC50)_competitor_ = (K_i_)_T4_/(K_i_)_competitor_

The relative potency for a competitor is defined as (IC50)_T4_/(IC50)_competitor_, and thus the above calculation can be re-arranged as:

(K_i_)_competitor_ = (K_i_)_T4_/(relative potency)_competitor_

Note that at low concentrations of radioligand, the K_i_ for a competitor approximates the corresponding IC50, which is the case herein.

## Method validation

**Fig. 3:**
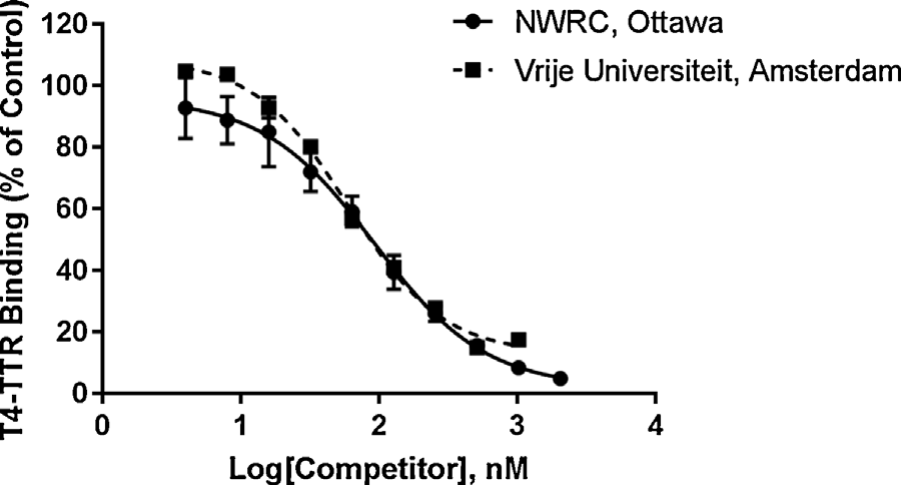
Competitive ligand binding curves showing the inter-laboratory reproducibility of thyroxine (T4)-transthyretin (TTR) calibration for results produced in our NWRC (Ottawa, Canada) laboratory (n = 6) compared to the results produced in the laboratory at the Vrije Universiteit Amsterdam (Amsterdam, The Netherlands) (n = 2). Results are presented as relative percent of T4 bound to TTR compared to controls (means ± standard deviations).

A method calibration curve should be generated in duplicate each time an assay batch is performed, using the series of prepared concentrations of the natural ligand as the competitor (e.g., 4 to 2048 nM T4 for TTR, plus negative controls). To ensure instrument performance, the gamma counter should be calibrated prior to each use, and instrument blanks (i.e., empty test tubes) can be included at various points during measurement of sample radioactivity. Serving as a positive control, each assay should include triplicate samples with the IC50 concentration of a known potent competitor. For example, the IC50 of 4-OH-BDE-49 for TTR is 12.2 nM (Table 3); other potential competitor ligands could include 4-OH-CB-187 (CAS 158076-68-7) [9] and tetrabromobisphenol A (TBBPA, CAS 79-94-7) [1], [8]. Negative controls should also be included for each assay batch, where the competitive ligand is absent and replaced with only DMSO. An additional method validation test that can be included in the assay procedure is a test of column performance using a sample prepared as a negative control but containing Tris-EDTA buffer in place of TTR. With this control, the ligands are in an unbound state and therefore should remain in the column after centrifugation, resulting in background radioactivity in the eluate. All assays should include triplicate samples, and should be conducted twice on separate days to include inter-and intra-day replicates (n = 6 total). Inter-laboratory reproducibility of this *in vitro* assay was confirmed by comparing T4-TTR calibration data produced in our National Wildlife Research Centre (Ottawa, Canada) laboratory to those produced in the laboratory of Dr. Timo Hamers (Department of Environment & Health, Vrije Universiteit Amsterdam, Amsterdam, The Netherlands) (Fig. 3).
